# Redox Status Is the Mainstay of SARS-CoV-2 and Host for Producing Therapeutic Opportunities

**DOI:** 10.3390/antiox11102061

**Published:** 2022-10-19

**Authors:** Anand Thirupathi, Yaodong Gu, Zsolt Radak, Ricardo A Pinho

**Affiliations:** 1 Faculty of Sports Science, Ningbo University, Ningbo 315211, China; 2 Research Institute of Sports Science, University of Physical Education, 1123 Budapest, Hungary; 3 Laboratory of Exercise Biochemistry in Health, Graduate Program in Health Sciences, School of Medicine and Life Sciences, Pontificia Universidade Catolica do Parana, Curitiba 80215-901, PR, Brazil

**Keywords:** SARS-CoV-2, COVID-19, ROS, oxidative stress, redox homeostasis, antioxidants

## Abstract

Over hundreds of years, humans have faced multiple pandemics and have overcome many of them with scientific advancements. However, the recent coronavirus disease (COVID-19) has challenged the physical, mental, and socioeconomic aspects of human life, which has introduced a general sense of uncertainty among everyone. Although several risk profiles, such as the severity of the disease, infection rate, and treatment strategy, have been investigated, new variants from different parts of the world put humans at risk and require multiple strategies simultaneously to control the spread. Understanding the entire system with respect to the commonly involved or essential mechanisms may be an effective strategy for successful treatment, particularly for COVID-19. Any treatment for COVID-19 may alter the redox profile, which can be an effective complementary method for severe acute respiratory syndrome coronavirus 2 (SARS-CoV-2) entry and further replication. Indeed, redox profiles are one of the main barriers that suddenly shift the immune response in favor of COVID-19. Fortunately, several redox components exhibit antiviral and anti-inflammatory activities. However, access to these components as support elements against COVID-19 is limited. Therefore, understanding redox-derived species and their nodes as a common interactome in the system will facilitate the treatment of COVID-19. This review discusses the redox-based perspectives of the entire system during COVID-19 infection, including how redox-based molecules impact the accessibility of SARS-CoV-2 to the host and further replication. Additionally, to demonstrate its feasibility as a viable approach, we discuss the current challenges in redox-based treatment options for COVID-19.

## 1. Introduction

The selective life-forming elements in the periodic table lose or accept the electrons in earlier life formation which are said to be oxidized or reduced (redox reaction), as oxygen is the final electron acceptor in biology [1]. However, earlier redox reactions used methane and hydrogen as the primary life-forming molecules, which delayed ancient organisms from evolving to aerobic life until the rise of cyanobacteria-like microbes, which split water to produce oxygen [1,2]. Consequently, this scenario introduced oxygen as metabolic waste. Moreover, this produced partially oxidized intermediates such as superoxide, hydrogen peroxide, and hydroxyl radical, which are collectively called reactive oxygen species (ROS) and can cause damage to cellular components, including DNA, proteins, and lipids. Although this review focuses on the oxygen-related redox reaction and its physiopathological functions, ancient anaerobic life depended on sulfur redox chemistry. This generated more S/N hybrid species when interacting with reactive sulfur species (RSS) and reactive nitrogen species (RNS) [3,4], which put the sulfur-based metabolism at high risk for rapid oxidation. All these consequences led the organisms to have controlled electron transfer during the evolutionary process [5]. This occurred within the set point of redox potential inside the same cell at different sites [6]. Unilateral oxidation disrupts the redox process if it is not contained by specialized molecules called antioxidants. The employment of enzymatic and non-enzymatic antioxidants has restored the redox status of the cells, warranting the application of these molecules in treating various redox diseases [6].

**Figure 1:**
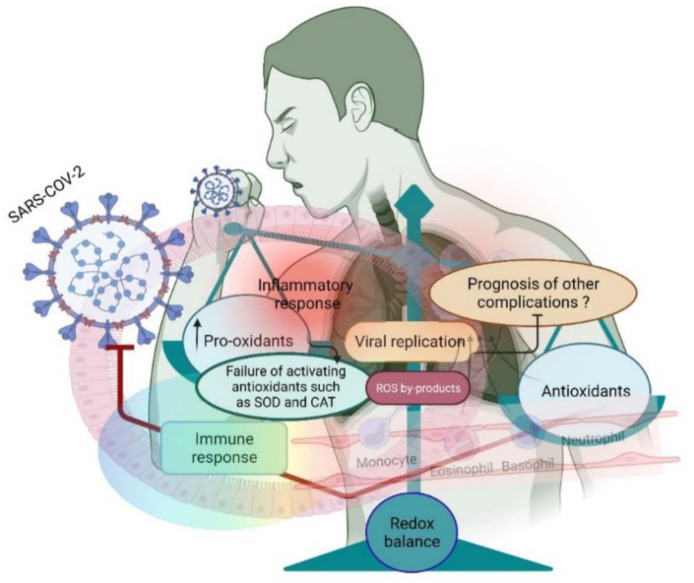
A small shift in the redox balance facilitates SARS-CoV-2 entry and further replication in the lungs. Entry of the virus induces an inflammatory response and increases the pro-oxidants to facilitate the host’s virus life cycle.

Although human populations have effectively overcome several pandemics, anthropogenic activities and the consequent impact on the ecosystem could increase the likelihood of other pandemics [7,8]. Indeed, the perplexities of the ecosystem alter microbial life more deeply than ever by changing the virulence and survival patterns of the microbes [9]. This is pertinent to the novel strain of severe acute respiratory syndrome coronavirus 2 (SARS-CoV-2), which caused a recent pandemic named “coronavirus disease 2019” (COVID-19) [10,11]. Accommodating human life to COVID-19 requires the synchronization of various molecules, including proteins, enzymes, and hormones that have been activated, modified or regulated by redox-active molecules [12]. Nevertheless, humans and microbes, including viruses, are symbiotically co-evolved; both may use these redox-active molecules for fundamental biochemical reactions that regulate cellular redox homeostasis, metabolism, signaling, and mitochondrial function. More ROS and increased oxidative stress disrupt redox-mediated cellular functions in favor of virus survival (Figure 1). For example, at the initial stage of any infection, including viral infection, the host system increases oxidative stress strategically to disrupt redox signaling; at the later stages, the host’s antioxidant system is activated to prevent damage [13]. However, the timeline of this scenario is a dilemma, suggesting that putting forward current knowledge gained in this field can help to establish underlying oxidative stress-induced pathophysiological mechanisms. Otherwise, proposing redox therapy as the most suitable one for COVID-19 may be a daunting challenge. In addition, the risk factors associated with COVID-19 commonly induce oxidative stress, suggesting that COVID-19 can be considered a redox disease [14]. This has been established with increased oxidative stress in COVID-19 patients [15]. However, it is difficult to recommend or formulate redox therapy for COVID-19, as every cell and tissue has a different redox setup in inducing oxidative stress (toward physiological functions) and oxidative damage (toward pathological conditions). For example, the redox potentials of NADH and NADPH are varied in the cytosol and mitochondria [16], which either induce oxidative stress or oxidative damage in the two compartments of the same cell. Therefore, this review addresses how redox-active molecules act as the mainstay of drawing up redox medicine for COVID-19.

Thus, the review starts with ROS sources during COVID-19. Identifying specific ROS sources may be a prognostic factor in determining COVID-19 disease severity. Next, this review will address how SARS-CoV-2 redesigns the redox system in the host in favor of its survival. This can prevent recommend unsuitable antioxidant therapy as it tilts the redox status. Finally, this review discusses all the challenges associated with oxidative stress-mediated pathophysiological mechanisms during COVID-19.

## 2. Sources of ROS during COVID-19

**Figure 2:**
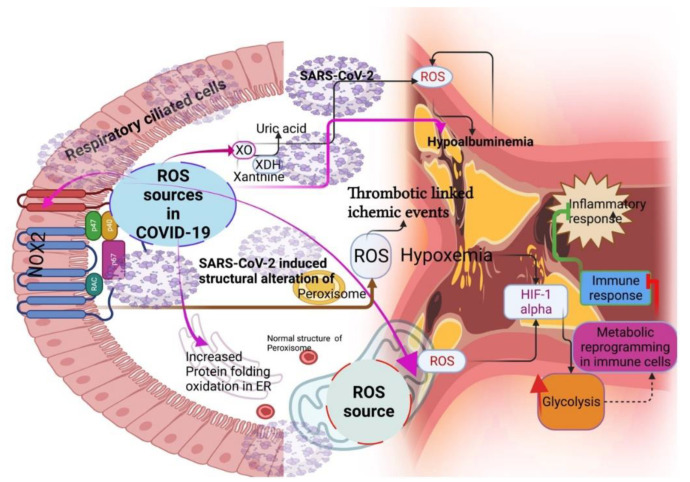
Sources of ROS during COVID-19 (pink arrow mark). NOX2-induced ROS increases the thrombotic-linked ischemic events in COVID-19. Mitochondrial-triggered ROS reprogram the metabolism for inhibiting immune response through hypoxia-inducible-1 alpha factor (HIF-1 alpha). SARS-CoV-2 altered the peroxisome morphology, consequently increasing ROS generation.

Respiratory viruses, including SARS-CoV-2, can induce ROS-generating sources such as nicotinamide adenine dinucleotide phosphate oxidases (NADPH oxidases) [17], xanthine oxidase (XO) [18], and mitochondria [19] (Figure 2). For instance, the activation of NOX2 and a consequent increase in ROS promote the setting of thrombotic-linked ischemic events in COVID-19 patients [20,21]. Although there is no direct evidence of XO as a source of ROS in COVID-19, a study showed that COVID-19 patients had hypouricemia (<2.5 mg/dl), which is implicated with the specific dysfunction of the proximal tubule [22]. As uric acid is an antioxidant, a decrease in uric acid might increase ROS and oxidative stress in the renal artery and further dysfunction in the kidney of COVID-19 patients [23], indicating the role of XO as an important source of ROS in COVID-19 [23]. Mitochondria are crucial sources of ROS, and mitochondrial-triggered ROS induces hypoxia-inducible-1 alpha factor (HIF-1 alpha) for improving glycolysis in the monocytes and macrophages [24]. This could directly inhibit the immune response, specifically, T-cell response [25], and decrease epithelial cell survival in COVID-19 [24]. Peroxisomes are another detoxifying source of ROS that have close contact with mitochondria as they are involved in the oxidative metabolism of amino acids and fatty acids [26]. Alterations in the peroxisome structure and loss of matrix content can increase ROS generation [27]. SARS-CoV-2 ORF14 protein altered the morphology of peroxisomes and their biogenesis by interacting with human PEX14 [28]. This could tilt the ROS balance in the host cells and increase lipid peroxidation, consequently inducing an inflammatory lipid storm in the lungs of COVID-19 patients by altering the leukotrienes and prostaglandins [29,30]. A double-center retrospective study reported that COVID-19 patients with severe conditions had hypoxemia [31], which may be from the ER stress response induced by ROS generation in the ER through protein-folding oxidation [32]. Notably, proteins such as serum albumin bind with several transition metals, such as iron and copper, called a “sponge” or a “tramp steamer” [33], to decrease ROS generation. The ligand-binding capacities of albumin are the reason for its antioxidant properties [33,34,35,36]. A low albumin level could increase the ROS level in COVID-19 patients, and retrospective studies have shown that hypoalbuminemia indicates the COVID-19 severity independent of age and co-morbidity [37,38].

## 3. SARS-CoV-2 Controls ROS Levels for Its Survival

An increase in ROS could cause damaging outcomes to SARS-CoV-2. To overcome this, viruses could naturally evolve to counteract ROS-induced oxidative damage in the cellular environment [39]. Possibly, this could increase the antioxidant capacity of the host to control the ROS environment. This concept was established with Huh7 cells, which underwent genetic reprogramming to permit hepatitis C virus (HCV) subgenomic replicon, induce oxidative stress by altering iron homeostasis, and activate manganese superoxide dismutase (MnSOD) and glutathione peroxidase 4 (GPx4) [40,41]. Regarding SARS-CoV-2, an observational study showed an increase in antioxidant capacity by inducing SOD and CAT in COVID-19 patients [42]. However, this was inconsistent with other COVID-19 patients [43]. This warrants additional studies to establish the link between SARS-CoV-2 and the host antioxidant system’s activation. Next, SARS-CoV-2 probably controls the excess ROS for survival through a metabolic switch. For example, SARS-CoV-2 may increase glycolysis to divert fuel to generate anabolic intermediates, similar to the Warburg effect [44,45]. This scenario prevents mitochondrial ROS. Supporting this idea, increased lactate dehydrogenase activity (LDH) was reported in COVID-19 patients [45]. Additionally, the knockdown of LDH increases pyruvate and promotes oxidative stress [46], suggesting that SARS-CoV-2 controls ROS using metabolic shifts [37]. Establishing this concept could form a new therapeutic approach to support redox-based treatment options. Next, SARS-CoV-2 induces mitochondrial dysfunction by decreasing mitochondrial membrane potential and causing mitochondrial permeability transition pore opening (MPTP), resulting in increased ROS release [47,48]. Studies have shown that viruses can induce apoptosis mechanisms for tissue injury or disease progression [49,50,51]. For example, SARS-CoV-2 ORF3a can induce an extrinsic apoptotic pathway by activating/cleaving caspase-8 [52]; targeting SARS-CoV-2-induced apoptosis could offer a promising target for SARS-CoV-2 treatment. Furthermore, SARS-CoV-2 fabricates intracellular signaling for survival, mainly through increasing H_2_O_2_. For instance, SARS-CoV-2 may decrease the accumulation of selenoprotein transcripts that regulate the phospholipid hydroperoxide glutathione peroxidase (GPX4) and mitochondrial functions [53], resulting in an increase in H_2_O_2_ in the host cells. This scenario alters redox-sensitive proteins such as mitogen-activated protein kinase (MAPK), signal transducer and activator of transcription proteins (STATs), Toll-like receptors (TLRs), nuclear factor kappa-light-chain-enhancer of activated B cells (NF-kB), and nuclear factor erythroid 2-related factor 2 (Nrf-2) [53]. In addition, the hyperactivation of TLR 3 and 4 could induce pro-inflammatory cytokines, including IL-1 and IL-6, and cause a cytokine storm for SARS-CoV-2 survival [54,55,56].

## 4. Redox Chemicals Coordinate Local and Systemic Redox Networks in COVID-19

**Figure 3:**
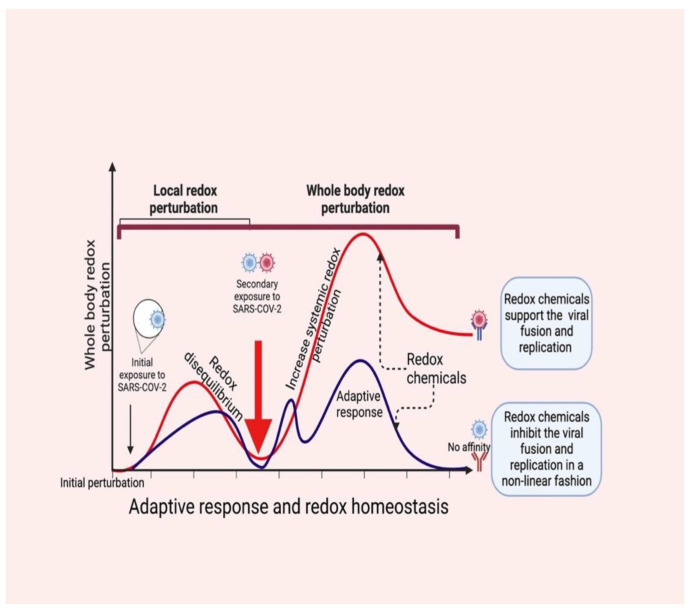
SARS-CoV-2 infection perturbs redox homeostasis in supporting viral fusion and replication (red color). Local redox disequilibrium causes systemic redox disequilibrium, which favors the entry of SARS-CoV-2 and consequent viral loads (secondary exposure). Redox molecules act as redox codes for proper redox communication, which induces an adaptive response by causing redox equilibrium in the host (blue color). This supports viral inhibition.

Redox-active molecules coordinate the systemic redox network (nonlinearly) locally first (beginning of the redox disequilibrium), and then turn to the entire system [57], which collapses the systemic redox equilibrium. Consequently, changes in the redox tones in the cells induce an “oxidative storm” in place of the “cytokine storm” [14], which could allow SARS-CoV-2 (probably a secondary exposure) to bypass redox-mediated immune vigilance (respiratory burst). This scenario can encourage virus fusion and viral loads to increase within the infection (Figure 3) [58,59].

**Figure 4:**
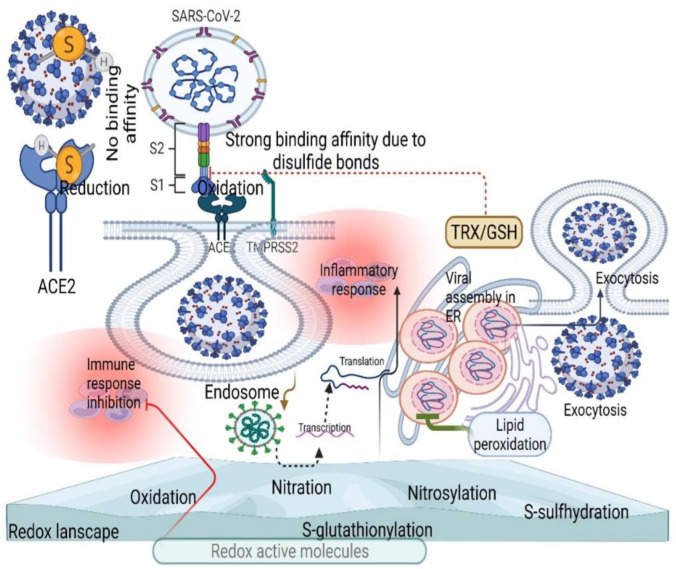
Changes in the redox landscape (thiol-based redox mechanisms) facilitate viral fusion and replication. Disulfide bonds induce a stronger affinity between SAS-COV-2 and ACE2, while thiol compounds do not induce binding affinity between SARS-CoV-2 and ACE2 (red inhibitory mark). Disulfide activation by the allosteric mechanism facilitates initial viral fusion by activating various redox molecules (endocytosis). In contrast, redox imbalance induces lipid peroxidation to inhibit viral replication in the ER (green inhibitory mark).

Although redox byproducts such as H_2_S, nitroxyl, beta-hydroxybutyrate, and different mixed sulfide compounds rebalance the entire redox network by acting as redox codes [4,59], active disulfides in the host contribute to spike protein binding and ACE2 by allosteric regulation (Figure 4) [60]. Perhaps GSH could negatively mediate this effective binding process by reducing active disulfides, which is confirmed by the decrease in GSH and increase in oxidative stress in severe COVID-19 patients [61,62], showing the therapeutic value of GSH in COVID-19. This scenario could also activate various redox molecules in the redox landscape, allowing the virus to enter the host cells by endocytosis, where ACE2 translocates to the endosomal lumen [63,64].

## 5. Antioxidant Therapy in COVID-19

The link between redox metabolism and viruses has been considered within the field of antioxidant intervention for several years. Although the purpose of recommending antioxidant therapy is not new, the recent pandemic brought antioxidant therapy to the frontline, as COVID-19 lacks specific antiviral drugs. N-acetyl cysteine (NAC) is considered to be the best-known antioxidant for alleviating SARS-CoV-2 infection [65]. There are some possible mechanisms proposed thus far, such as interfering with angiotensin II cleavage to angiotensin 1–7 via ACE2 and attenuating oxidative damage by increasing TLR-7, restoring type-IFN production [66], and antagonizing proteasome inhibitors to reduce the accumulation of the viral proteins [66,67]. NAC possibly interacts with the SARS-CoV-2 E protein by cleaving disulfide bonds of the triple cysteine motif (NH2- … L-Cys-A-Y-Cys-Cys-N … -COOH) [66]. This protein regulates the cellular polarity and cell–cell junctions in the epithelial cells by binding the PALS1 PDZ domain [67]. This could reduce the infection rate. NAC prevents the glycosylation events in SARS-CoV-2 by restoring platelet GSH, suggesting an alteration in the GSH level could be a therapeutic approach for fighting against SARS-CoV-2 [67,68]. Higher activation of proinflammatory cytokines is common in patients with a level of TNF-alpha [69]. Therefore, regulating these molecules ensures the reinstatement of the immune system. Antioxidant therapy could be the major antagonist to these pro-inflammatory cytokines which alleviate hyperinflammation in patients with severe COVID-19 [70]. A recent in vitro *and* ex vivo study reported that nanocoated CAT downregulates pro-inflammatory cytokines to regulate immune homeostasis [71]. However, these methods are unreliable in assessing ROS and oxidative damage, as they are vulnerable to artifacts. Nitric oxide (NO) has another potential molecule that inhibits SARS-CoV-2 replication and enhances oxygenation in COVID-19 patients. The administration of H_2_S inhibits oxidative stress by preventing platelet activation and neutrophil extracellular trap (NET) formation. The ingestion of H_2_S may be an effective treatment for COVID-19. Other antioxidants, including CoQ10 with NADH, curcumin, vitamins C, D, and E, selenium, melatonin with pentoxifylline, ebselen, and disulfiram, can target redox imbalance in COVID-19 [72,73,74,75,76,77,78,79]. For example, the use of antioxidants such as vitamin C, E, and NAC with pentoxifylline decreased the lipid peroxidation and total antioxidant capacity in COVID-19 patients at the end of the hospital stay, while pentoxifylline alone did not decrease the oxidative stress markers [80], suggesting the use of antioxidants as a possible adjuvant therapy for improving survival prognosis in COVID-19 patients. However, further studies are warranted to prove the reproducibility of this data. Because noncatalytic cysteine residues of the 3C-like protease (3CL^pro^) in SARS-CoV-2 can protect the virus from oxidative damage, as current drugs such as nirmatrelvir are mainly inhibiting the cysteine residue of 3CL^pro^, the use of these antioxidants may interfere with this process and aggravate SARS-CoV-2 replication.

## 6. Challenges Associated with Oxidative Stress during COVID-19 Treatment

Although various vaccines and drugs are a major part of treatment support during COVID-19, the risk profile of patients is ambiguous. Rapidly spreading variants from different parts of the world is an additional concern that effectively overcomes the available treatments [81,82]. Furthermore, targeting localized oxidative damage (specific tissue environment) with antioxidants perturbs the systemic redox network [14]. Furthermore, treatment options, either with vaccines or drugs, perturb redox homeostasis and induce oxidative stress. For example, tocilizumab and hydrocortisone provide better protection against COVID-19 [83]. However, these drugs with ventilatory support induce oxidative damage and alter the immune response while protecting the endothelial glycocalyx, whose function is to maintain the redox balance in COVID-19 [83]. Dexamethasone is another drug that can activate redox-active molecules and increase oxidative stress, enabling antioxidant defense through KEAP1/NRF-2 activation, affecting electron transport complexes, and increasing NOX-2, all of which dominate the redox system [84,85]. Capivasertib could inhibit SARS-CoV-2 entry by improving glycolysis and oxidative phosphorylation through ROS-mediated AKT inhibition [86]. Hydroxychloroquine and ivermectin can also induce ROS production in vitro and in vivo [85,87,88]. Other drugs, such as anakinra, sotrovimab, and ruxolitinib exacerbate ROS pathways to control COVID-19 [87,88]. Treatment with these drugs increases oxidative stress and compromises their clinical efficacy for COVID-19 treatment [89]. Therefore, implementing redox-based therapies requires a deeper knowledge of oxidative stress research. However, recent advancements in this field, from redox imaging to redox metabolomics, will facilitate the revealing of the key molecule’s functions in biological systems. Furthermore, advanced omics methods can support characterizing redox molecules and their functions. This will provide an opportunity to overcome current challenges associated with COVID-19 treatment. Otherwise, using nonspecific kits to measure oxidative damage will not provide any definite results to treat COVID-19, because using these kits can partially reflect the oxidative stress status within the cells. For instance, ROS probes such as 2′,7′-dichlorodihydrofluorescein (DCFH) can be oxidized with several ROS molecules and are not specific for any particular ROS [90]. Therefore, monitoring real-time changes in the intracellular redox-active molecules will help to develop a high-throughput global profiling methodology for finding ROS molecules.

## 7. Conclusions

This review discussed several ROS sources that are perhaps activated by SARS-CoV-2 for its survival. This may increase local and systemic oxidative stress. However, why this scenario does not affect SARS-CoV-2 survival in the host is unknown. Instead, it can escape from the host oxidative stress response using its noncatalytic cysteine residues. Using any currently available drugs with redox-based adjuvant therapies or as a main therapy may interfere with this cysteine-mediated oxidation and could support SARS-CoV-2 replication. Therefore, careful evaluation is required to design an integrated approach to understand the whole body’s systemic redox status. From this perspective, we have discussed the possible reasons for this scenario, starting from ROS sources to therapeutical challenges in treating COVID-19. This may help to consider COVID-19 as a redox disease and support redox-based therapies as the possible preference for COVID-19 treatment. However, before implementing redox-based therapies, every COVID-19 patient should be interrogated with oxidative stress parameters, as they can show different redox statuses. Moreover, personalized approaches will require appropriate redox-based intervention, as redox homeostasis is more susceptible to a specific intervention, which further provokes oxidative stress. Consequently, disconnecting the redox-mediated communication between the organs can reduce the chances of regaining COVID-19 control. Therefore, the routine collection of clinical information with regard to the redox status of an individual may provide new therapeutic opportunities to restore the redox balance and decrease COVID-19 mortality and possibly manage long-COVID symptoms.
